# Supplementary material for: Splice-Junction-Based Mapping of Alternative Isoforms in the Human Proteome
Source: Cell Rep. Author manuscript; Available in PMC 2020 Jan 15. (PMC6961840; doi:10.1016/j.celrep.2019.11.026)
Supplement: 2 [file NIHMS1546469-supplement-2.zip › DF1/PXD006675/LeftCoronaryArtery/LeftCoronaryArtery_16_TRIM39_NAAPLTIRPPTDWDVK.pdf]

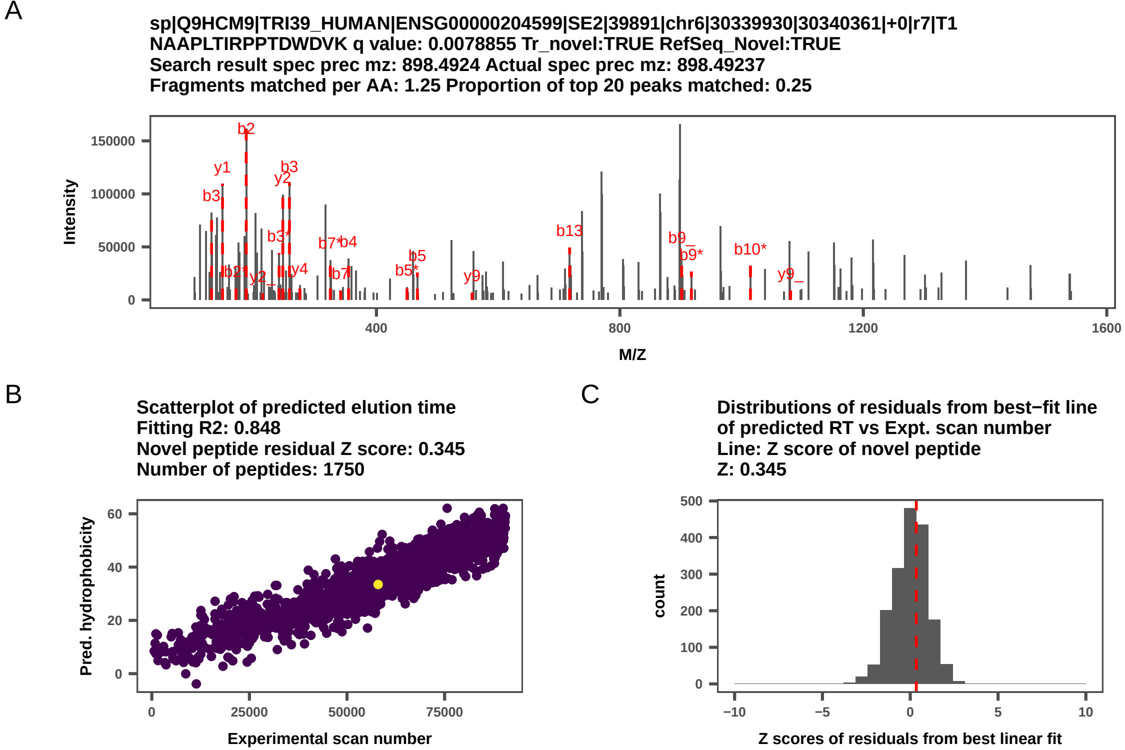
A
sp|Q9HCM9|TRI39_HUMAN|ENSG00000204599|SE2|39891|chr6|30339930|30340361|+0|r7|T1
NAAPLTIRPPTDWDVK q value: 0.0078855 Tr_novel:TRUE RefSeq_Novel:TRUE
Search result spec prec mz: 898.4924 Actual spec prec mz: 898.49237
Fragments matched per AA: 1.25 Proportion of top 20 peaks matched: 0.25
Intensity
150000
100000
50000
0
M/Z
400
800
1200
1600
b2
y1
b3
y2
b3
b3*
b2*
y2_
y4
b7*b4
b7
b5
b5*
y9
b13
b9_
b9*
b10*
y9_
B
Scatterplot of predicted elution time
Fitting R2: 0.848
Novel peptide residual Z score: 0.345
Number of peptides: 1750
Pred. hydrophobicity
60
40
20
0
0
25000
50000
75000
Experimental scan number
C
Distributions of residuals from best−fit line
of predicted RT vs Expt. scan number
Line: Z score of novel peptide
Z: 0.345
count
500
400
300
200
100
0
−10
−5
0
5
10
Z scores of residuals from best linear fit
